# Physical Activity and Body Composition in Children and Their Mothers According to Mother’s Gestational Diabetes Risk: A Seven-Year Follow-Up Study

**DOI:** 10.3390/medicina55100635

**Published:** 2019-09-25

**Authors:** Marja H Leppänen, Jani Raitanen, Pauliina Husu, Urho M Kujala, Pipsa PA Tuominen, Henri Vähä-Ypyä, Riitta Luoto

**Affiliations:** 1 Faculty of Sport and Health Sciences, University of Jyväskylä, 40100 Jyväskylä, Finland; urho.m.kujala@jyu.fi (U.M.K.);; 2 The UKK Institute for Health Promotion Research, Kaupinpuistonkatu 1, 33500 Tampere, Finland; 3 Faculty of Social Sciences (Health Sciences), Tampere University, 33014 Tampere, Finland; 4 Faculty of Medicine and Health Technology, Tampere University, 33014 Tampere Finland; riitta.luoto@fimnet.fi

**Keywords:** exercise, gestational diabetes risk factors, pediatrics, body composition, follow-up

## Abstract

*Background and Objectives:* There is lack of knowledge on whether mothers’ gestational diabetes (GDM) risk is related to their physical activity (PA) or their children’s PA and body composition. We aimed to examine the difference in (1) change in self-reported PA from pre-pregnancy to seven-year follow-up and (2) accelerometer-based PA at seven years after delivery between the mothers having GDM risk (GDMRyes-mothers) and not having GDM risk (GDMRno-mothers). Furthermore, we examined the difference in children’s PA and/or body composition at six years of age according to their mothers’ GDM risk. *Materials and Methods:* The study included 199 Finnish women. GDM risk factors were screened at the beginning of pregnancy, and the women were classified as GDMRyes-mothers if they had at least one GDM risk factor (body mass index ≥25 kg/m^2^; age ≥40 years; family history of diabetes; GDM, signs of glucose intolerance, or newborn’s macrosomia (≥4500 g) in earlier pregnancy) or as GDMRno-mothers if they had no risk factors. Mothers’ PA was assessed by self-reporting at 8–12 gestational weeks concerning pre-pregnancy PA and at a follow-up seven years after the delivery. Moreover, mothers’ and their children’s PA was measured using a triaxial Hookie AM20-accelerometer at seven years after delivery. Children’s body composition was assessed using a TANITA bioelectrical impedance device. Adjusted linear regression analyses were applied. *Results:* GDMRno-mothers increased their self-reported PA more than GDMRyes-mothers from pre-pregnancy to the seven-year follow-up. Concerning women’s measured PA as well as children’s PA and body composition at seven years after delivery, the differences were non-significant between GDMRyes-mothers and GDMRno-mothers. However, of the GDM risk factors, mothers’ pre-pregnancy body mass index was positively related to unhealthier body composition in boys at six years of age. *Conclusion:* Health promotion should be targeted at women with GDM risk factors, in particular overweight women, in enhancing women’s PA in the long term and their children’s healthy body composition.

## 1. Introduction

Gestational diabetes (GDM) is a complication of pregnancy that is associated with higher risk of adverse health outcomes for both the mother and offspring [1]. The prevalence of GDM is increasing worldwide [2], and in Finland, 12.6% of women were diagnosed with GDM in 2016 [3]. Well-recognized risk factors for GDM are high maternal age, overweight, family history of type 2 diabetes, and GDM or glucose intolerance in previous pregnancies [4]. In 2015, pregnant women’s body mass index (BMI) was on average 24.7 kg/m^2^ in Finland [3]. However, a long-term influence of the GDM risk factors on the mothers and/or their children has been less studied.

Regular physical activity (PA) is highly recommended throughout the lifespan due to its numerous health benefits [5], such as a reduced risk of GDM development during pregnancy [6]. Sedentary behavior (SB), however, should be reduced due to its many health risks [5]. The PA recommendation for all healthy women during pregnancy and postpartum (the year following the baby’s birth) was set by the American College of Obstetricians and Gynecologists [6]. However, women rarely meet the recommendation during pregnancy [7] or during the postpartum period [8,9]. PA tends to decrease during pregnancy [7,10] and to increase again after the delivery, although still remaining below the pre-pregnancy level [8,11]. Pregnancy and the postpartum period are ideal times for adopting a healthy lifestyle due to increased motivation and frequent access to medical supervision. Yet, there is a lack of knowledge about the factors related to PA from pre-pregnancy over a longer period. Such information would help health care professionals to offer interventions to the right target groups.

PA in early childhood (under seven years of age) has been connected to health indicators, such as lower adiposity [12,13], and hence, defining the factors beyond PA is needed. Maternal pre-pregnancy overweight was previously found to be associated positively with childhood obesity [14]; yet, due to the high prevalence of GDM risk factors in pregnant women, it is essential to investigate whether the GDM risk has an influence on children’s PA and/or body composition.

Since women are sensitive for behavior modifications during pregnancy, the “teachable moment” in promoting long-term health-enhancing PA in mothers and their offspring might be beneficial to use. Thus, we sought to investigate the difference in (1) change in self-reported PA from pre-pregnancy to a seven-year follow-up and (2) accelerometer-based PA at seven years after delivery between the mothers having at least one GDM risk factor (GDMRyes-mothers) and those not having GDM risk (GDMRno-mothers). Moreover, we investigate whether there is a difference in children’s PA and/or body composition at six years of age according to their mothers GDM risk factor status.

## 2. Materials and Methods

### 2.1. Study Design and Participants

This study utilized baseline and follow-up data of the Finnish NELLI seven-year follow-up study. The baseline data is based on a cluster-randomized controlled trial aiming to prevent GDM (NELLI) [15], and the effects of the intervention on GDM [16] and PA [17] have been previously reported. All participants provided written informed consent, and The Pirkanmaa Ethics Committee in Human Sciences provided an ethical statement for the randomized controlled trial (RCT) (code R06230, 19.1.2007) and follow-up studies (code R14039, 8.10.2014).

**Figure 1:**
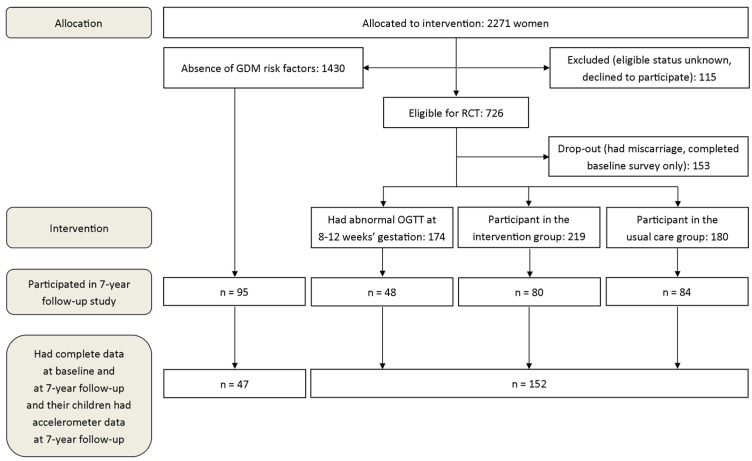
Flow chart of the study.

Figure 1 shows the flow chart of participants. Pregnant women with at least one GDM risk factor, were permitted to enter the RCT study; however, the women without GDM risk factors were also allowed to respond to the PA questionnaire at baseline. The risk of GDM was screened by using the following risk factors: BMI ≥ 25 kg/m^2^; age ≥40 years; family history of diabetes; GDM or any signs of glucose intolerance or newborn’s macrosomia (≥4500 g) in any earlier pregnancy. The exclusion criterions were reported elsewhere [15].

Women were included in this study if they had responded to the PA questionnaire at baseline and at follow-up, and if they as well as their children had at least four days of accelerometer-based PA at seven years after delivery. In total, 199 dyads provided all the required data and they were included to the study. Of the 199 women, 56 (28.1%) were in the intervention group in the RCT study, 52 (26.1%) were in the usual care group, 32 (16.1%) were excluded from the RCT study due to GDM, and 59 (29.6%) were excluded from the RCT due to the lack of GDM risk factor.

### 2.2. Data Collection

Self-reported physical activity. PA was assessed with a validated self-report at two different time points. Pre-pregnancy PA was assessed at 8–12 gestational weeks using the questions that concerned PA during a typical week prior to pregnancy. PA at the seven-year follow-up was assessed at seven years after the delivery using the questions that concerned PA during the past one month [18,19]. The intensity-specific PA levels were categorized as light PA (LPA), moderate PA (MPA), and vigorous PA (VPA), and further, MPA and VPA were merged as moderate-to-vigorous PA (MVPA) and all PA intensities calculated as totalPA (TPA).

Accelerometer-based physical activity. At seven years after delivery, data on PA and SB were collected with a triaxial Hookie AM20-accelerometer (Traxmeet Ltd., Espoo, Finland), which was shown to be a valid measurement tool among adults [20] and adolescents [21], and a feasible device among preschool children [22]. The mothers and their children wore the hip-worn device for seven consecutive days during waking hours, except when showering or if they had other water activities. Intensity of PA was estimated using mean amplitude deviation (MAD) of acceleration analyzed in 6 s epochs [20]. The MAD value of each epoch was converted to metabolic equivalent (MET) value. The cut-points were defined for SB as <1.5 MET, for LPA as 1.5–2.9 MET, for MPA as 3.0–5.9 MET, and for VPA as ≥6.0 MET. A valid day was defined as ≥10 h of awake wearing time. Some participants decided to sleep with the accelerometer, in which case the measurement time was over 18 h per day, and thus, the exceeding time was taking off from the SB time.

Maternal demographic characteristics. Pre-pregnancy weight and height were based on a self-report, and on measurements at the seven-year follow-up. Data on age, parity, education and working status at the first maternity clinic visit and at the seven-year follow-up were obtained from the standard maternity card and a questionnaire.

Children’s anthropometry and body composition. At seven years after delivery, height was measured using a wall stadiometer, while weight and body composition were measured using the TANITA bioelectrical impedance analysis (MC-780MA, TANITA Corporation, Tokyo, Japan). The body composition measures included were BMI, fat mass index (FMI), fat-free mass index (FFMI), and fat mass percent (FM%). BMI was calculated as body weight (kg)/height^2^ (m), and it was classified in accordance with the Finnish reference data [23]. FMI was calculated as fat mass (kg)/height^2^ (m), and FFMI as fat-free mass (kg)/height^2^ (m).

### 2.3. Statistical Methods

Descriptive information is presented as means ± standard deviation (SD) or frequencies (%). The women were stratified into two groups: GDMRyes-mothers and GDMRno-mothers. Linear regression analysis was used to examine the difference between the groups in (1) change in self-reported PA from pre-pregnancy to the seven-year follow-up, and (2) accelerometer-based PA and SB at seven years after delivery. Each model was adjusted for the child’s age and BMI, parental education, working status, and number of children under seven years in the home as well as for baseline PA (change in self-reported PA) and awake wearing time of the accelerometer (measured PA). Since part of the women participated in the RCT, we investigated whether there were differences on PA outcomes by categorizing the exposure variables in four groups: (1) intervention or (2) usual care group in the RCT, (3) excluded from the RCT due to GDM, and (4) excluded from the RCT due to the lack of GDM risk factor. Since the results did not differ essentially, we decided to present the results in two groups as mentioned above.

The differences in children’s PA, SB, and body composition according to their mothers’ GDM risk status, were examined using a *t*-test (continuous variables) and Chi-square test (categorized variables). In supplementary analyses, we used linear regression models to investigate the associations of maternal pre-pregnancy BMI and children’s measured PA, SB, and body composition at six years of age. Due to the significant interaction between maternal pre-pregnancy BMI and the child’s sex on the body composition outcomes, girls and boys were investigated separately. We adjusted the models for the child’s age, maternal education (categorized), and the models concerning PA for the awake wearing time of the accelerometer.

For all analyses, a *p*-value of <0.05 was considered statistically significant. Statistical analysis was performed using SPSS Statistics 24 (IBM, Armonk, NY, USA).

## 3. Results

The demographic characteristics of the participating mothers and children, stratified according to the GDMRno-mothers (n = 47) or GDMRyes-mothers (n = 152) at baseline, are presented in Table 1.

### 3.1. Change in Self-Reported PA between Baseline and Seven-Year Follow-Up

**Figure 2:**
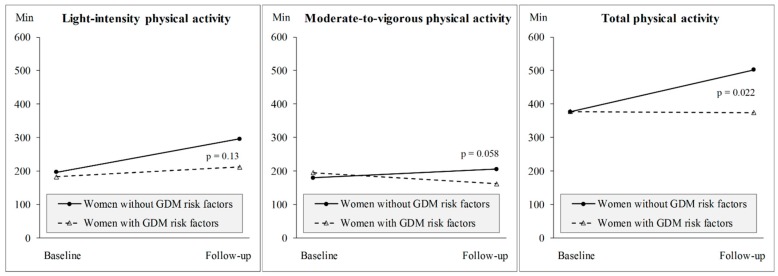
The weekly minutes of change in self-reported intensity-specific physical activity from baseline (pre-pregnancy) to the seven-year follow-up stratified by the women with (n = 152) or without (n = 47) gestational diabetes (GDM) risk. *p*-values represents the significance of the difference in change in physical activity at the seven-year follow-up between the groups.

At baseline (pre-pregnancy), among GDMRno-mothers, the weekly minutes of self-reported LPA were on average 197, MVPA 180, and TPA 377, while the corresponding minutes among GDMRyes-mothers were on average 183, 195, and 378 (Figure 2). Among both GDMRno-mothers and GDMRyes-mothers the most frequently reported types of PA prior to pregnancy were walking (12–93% by intensity), jogging/running (5–31%), and instructed classes (4–22%). GDMRno-mothers had LPA on average 3.5 (SD 2.5) times per week, and MVPA 4.1 (SD 2.4) times per week, while the corresponding times per week among GDMRyes-mothers were 4.8 (SD 2.2) and 3.9 (SD 2.5), respectively. There were no significant differences in PA times per week between GDMRno-mothers and GDMRyes-mothers (*p* > 0.05).

Furthermore, the weekly minutes at the seven-year follow-up were on average 296, 206, and 502 among GDMRno-mothers, and 212, 162, and 374 among GDMRyes-mothers, respectively. The differences were significant between GDMRno-mothers and GDMRyes-mothers regarding LPA (*p* = 0.012) and TPA (*p* = 0.003) at the seven-year follow-up, and borderline regarding MVPA (*p* = 0.051). Further, the change in TPA between baseline and seven-year follow-up was significantly different (Figure 2). Among GDMRno-mothers the most frequently reported types of PA at the seven-year follow-up were walking (43–51% by intensity), jogging/running (9–40%) and playing with children (6–32%), while among GDMRyes-mothers the most frequently reported types of PA were walking (37–54%), gym training (11% in MPA), and playing with the children (6–22%). GDMRno-mothers had LPA on average 4.8 (SD 2.2) times per week and MVPA 4.2 (SD 2.7) times per week, while the corresponding times per week among GDMRyes-mothers were 3.7 (SD 2.2) and 3.3 (SD 2.1).

GDMRyes-mothers had significantly less LPA times per week compared to GDMRno-mothers (*p* = 0.006).

The adjusted model indicates that GDMRno-mothers had on average an 88 min/week higher change in LPA compared to GDMRyes-mothers (*p* = 0.012) (Table 2). In addition, GDMRno-mothers had on average 130 min/week higher change in TPA compared to GDMRyes-mothers (*p* = 0.004). Older age and not having had previous children were related to lower change in LPA and TPA over the seven years of follow-up (Table 2).

### 3.2. Mothers’ Measured PA at the Seven-Year Follow-Up

GDMRno-mothers and GDMRyes-mothers had on average 6.7 valid measurement days (SD 0.74 and 0.56). GDMRno-mothers spent on average 71% of their time in SB, 19% in LPA, and 10% in MVPA. GDMRyes-mothers spent 74% in SB, 17% in LPA, and 9% in MVPA, respectively. Table 3 shows that after adjusting for confounders, the differences were non-significant between GDMRno-mothers and GDMRyes-mothers. Current BMI, working status, and baseline education level, however, were related to time spent in PA levels (Table 3).

### 3.3. Children’s Measured PA and Body Composition

The children of the GDMRno-mothers (n = 47) had on average 6.6 valid measurement days (SD 0.68), and they spent on average 59% of their time in SB, 23% in LPA, and 18% in MVPA. The children of the GDMRyes-mothers (n = 152) had on average 6.5 valid days (SD 0.78), and their corresponding times in different PA intensities were 58%, 23%, and 19%, respectively. The differences between the groups were non-significant.

The children of the GDMRno-mothers had on average BMI 16.0 kg/m^2^, FM% 21.1, FMI 4.2 kg/m^2^, and FFMI 15.5 kg/m^2^. The corresponding means among the children of GDMRyes-mothers were 16.2 kg/m^2^, 22.1%, 4.4 kg/m^2^, and 15.4 kg/m^2^; yet, the differences were non-significant between the groups.

Boys spent more time in MVPA compared to girls (20% vs. 18%, *p* < 0.001); furthermore, boys had lower FM% compared to girls (21.3% vs. 22.5%, *p* = 0.045).

In supplementary analyses, each additional unit of maternal pre-pregnancy BMI increased boys’ BMI by 0.18 kg/m^2^ (*p* < 0.001), FM% by 0.29 (*p* = 0.001), FMI by 0.13 (*p* < 0.001), and FFMI by 0.12 (*p* = 0.002) at seven years after delivery. There were no significant relationships between any other pre-pregnancy GDM risk factors to children’s PA or body composition at the age of six.

## 4. Discussion

Mothers in the GDMRno-group significantly increased their self-reported LPA and TPA from pre-pregnancy to the seven-year follow-up compared to GDMRyes-mothers. Of the GDM risk factors, pre-pregnancy BMI was positively related to boys’ adiposity at 6 years of age. 

### 4.1. Change in Self-Reported PA between Baseline and Seven-Year Follow-Up

Despite of the amount of pre-pregnancy PA, GDMRno-mothers increased self-reported LPA and TPA over the seven-year period until the follow-up significantly more compared to GDMRyes-mothers. GDMRno-mothers may have an overall healthier lifestyle and be generally more physically active. Previously, it was reported that women with GDM were able to increase their self-reported PA from pre-pregnancy to one-year follow-up after the diagnosis, whereas the non-GDM group did not [24]. However, the findings are not comparable due to the methodological differences.

The findings highlight the need to target PA interventions to GDMRyes-mothers in reducing the risk of GDM [25] and excessive weight gain during pregnancy [26].

GDMRno-mothers increased their self-reported MVPA more over the seven-year follow-up period compared to GDMRyes-mothers when baseline PA was taken into account; yet, the difference was non-significant. It is possible that the number of GDMRno-mothers (n = 47) may not be big enough to indicate the statistically significant difference. Furthermore, the wide range in standard deviation probably affected the results. However, the finding of the current study is relevant, since BMI was observed to influence the difference between absolute PA intensities and PA intensities in relation to the individual physical fitness level [27]. It is possible that overweight women have had a similar reported MVPA level over the seven-year follow-up period as the normal weight counterparts, even if the absolute MVPA levels may have been different (we did not measure this). This outlook is important when promoting PA in overweight women, and it highlights the need to modify the recommended PA to individual fitness.

Age and not having had previous children were negatively related to change in self-reported LPA and TPA. The findings are somewhat expected since in our previous study [19], having a child was found to encourage women to be physically active during pregnancy. Furthermore, tiredness, nausea, and poor perceived health were reported to restrict women’s PA during pregnancy. Older women may feel more tiredness and their perceived health may be weaker, which may partly explain the result in this study.

### 4.2. Mothers’ Measured PA at the Seven-Year Follow-Up

There were no significant differences between GDMRyes-mothers and GDMRno-mothers. Since, out of 199 women, 152 (76.4%) had at least one GDM risk factor and, additionally, 51 women (33.6%) were diagnosed with GDM during pregnancy, the finding of this study is promising. The result suggests that whether the women have a GDM risk at the beginning of pregnancy or they are diagnosed with GDM during pregnancy, their PA and SB may be on the same level with GDMRno-mothers in the long-term. However, as common in research, the most active women were probably the ones who agreed to participate in the study, which may have affected the results.

Self-reported PA investigated longitudinal changes in PA over the seven-year follow-up, while accelerometer-based PA examined cross-sectional relationships at seven years after delivery. A self-reported questionnaire assessed PA during leisure-time and household activities, whereas occupational PA was not taken into account. Accelerometer-based PA, however, stored all PA during the waking hours. At the seven-year follow-up, self-reported TPA was significantly higher among GDMRno-mothers compared to their peers, but the difference was not seen in accelerometer-based PA at the same time point. This may partly be explained by the fact that accelerometer-based PA included also occupational PA and significantly more women at GDMRyes-mothers were working fulltime compared to their counterparts. Although the different assessments of PA are not comparable, it is noticeable that some of the results are consistent. In unadjusted models, GDMRno-mothers had significantly higher self-reported LPA and TPA at the seven-year follow-up compared to GDMRyes-mothers (Figure 2), and similar differences were seen in accelerometer-based LPA and SB (reflecting contradictory TPA) (Table 3).

### 4.3. Children’s Measured PA and Body Composition

There were no significant differences in children’s measured PA, SB, or body composition according to the GDM risk factor status of their mothers. All participating mothers may have received “a natural course”, indicating that all mothers were motivated to promote a healthy lifestyle in their children.

We are not aware of previous studies investigating the relationships according to GDM risks. A multinational cross-sectional study [28] found a positive association between diagnosed GDM and children’s obesity, but the association was not independent of maternal current BMI. Similarly, a small cohort study (n = 40) [29] reported a positive association between diagnosed GDM and obesity in children, but the association disappeared after adjusting for pre-pregnancy BMI. Thus, it seems that maternal pre-pregnancy and/or current BMI have a larger role in predicting childhood obesity than diagnosed GDM or the other GDM risk factors, and that may partly explain the lack of difference between the two groups in this study.

We found a significant association between mothers’ pre-pregnancy BMI with all body composition measurements (BMI, FM%, FMI, and FFMI) in boys. The finding is in line with previous studies in young children [14,30], except the lack of significant relationships in girls. The somewhat inconsistent findings may be partly explained by different study designs as well as participants’ different ethnic and genetic backgrounds. Shared genetic factors may have contributed to the association between maternal pre-pregnancy BMI and body composition in boys. Examining the protective effect of PA or dietary habits with body composition is challenging in young children due to the lack of long-term randomized controlled trials and methodological limitations of longitudinal observational studies [31]. Nevertheless, it seems that pre-pregnancy BMI may influence children’s body composition more in long-term than the other GDM risk factors.

There were no statistically significant associations between maternal pre-pregnancy BMI and children’s measured PA or SB. There are most likely other factors, such as parental PA and SB [32] and education [33], that influence children’s PA more. Since a negative association between maternal current BMI and children’s PA [33,34], as well as a positive association with children’s SB [34], were previously reported, it is possible that the mother’s current BMI influences children’s PA more at early ages than pre-pregnancy BMI. Furthermore, it is likely that overnutrition is also related to a high pre-pregnancy BMI influencing children’s adiposity rather than PA. It was reported that a physically active lifestyle of the family increases children’s PA [34], and therefore, there is a need for future studies to clarify the association between maternal pre-pregnancy BMI and children’s PA enabling the use of the “teachable moment” during pregnancy.

### 4.4. Strengths and Limitations

To our knowledge, this is the first study comparing the GDM risk factor status to the mothers’ PA and SB as well as to their children’s PA, SB, and body composition. The major strength of the study is the somewhat long follow-up period, compared to previous studies using follow-up periods of up to 12 months [8,24]. Additionally, PA and SB were measured using a validated accelerometer [20,21,22], which provided accurate information on PA and SB in both mothers and children. Furthermore, they had on average 6.5–6.7 valid days of accelerometer data per week increasing the reliability of the measured PA and SB.

The primary weakness of our study is that 28% of GDMRyes-mothers received a PA and dietary counseling during pregnancy. However, the counseling was not reported to be effective in increasing the weekly duration of PA [17] or reducing maternal GDM [16]. Nevertheless, the result did not differ essentially when analyses were adjusted for counseling. Secondly, self-reported information on PA is susceptible for misreporting. However, we used the same questionnaire at both time points, and therefore misreporting was most likely consistent. Finally, self-reported pre-pregnancy weight may have been underestimated, which may have led to underestimation of pre-pregnancy BMI. Due to that, the actual number of overweight participants may be higher.

The findings of the current study can be generalized to the target group due to the population-based recruitment and a high participation rate in the preliminarily eligible women (88%) [17]. However, it is possible that the participating women were physically more active, and in addition, more aware of healthier lifestyle. This may have influenced the results.

## 5. Conclusions

In conclusion, GDMRno-mothers increased their self-reported LPA and TPA from pre-pregnancy to seven-year follow-up more compared to GDMRyes-mothers. Of the GDM risk factors, pre-pregnancy BMI was positively related to boys’ adiposity at six years of age. Thus, health promotion should be targeted to the women with GDM risk factors, in particular overweight women, in order to enhance women’s PA in long-term, and their children’s healthy body composition.

## Figures and Tables

**Table 1:** Characteristics of the participants stratified by mothers’ gestational diabetes (GDM) risk status: (a) prior to pregnancy (baseline) and (b) at seven years after the delivery (follow-up).

	GDMRno ^a^ (N = 47)	GDMRyes ^a^ (N = 152)	*p*-Value for Difference
**Characteristics at baseline**			
Age (years)	29.3 (5.5)	30.2 (4.6)	0.26 ^b^
Height (cm)	166.6 (6.0)	166.5 (6.6)	0.92 ^b^
Weight (kg)	61.8 (6.6)	74.2 (14.5)	<0.001 ^b^
Body mass index, BMI (kg/m^2^)	22.3 (2.0)	26.8 (4.9)	<0.001 ^b^
Education, N (%)			
Academic	13 (28.9)	44 (28.9)	0.96 ^c^
Polytechnic	19 (42.2)	61 (40.1)	
Basic or secondary school	13 (28.9)	47 (30.9)	
Working status, N (%)			
Fulltime	30 (63.8)	94 (61.8)	0.81 ^c^
Part-time, unemployed, student, maternity leave	17 (36.2)	58 (38.2)	
Marital status, N (%)			
Married	46 (97.9)	148 (97.4)	0.85 ^c^
Not married	1 (2.1)	4 (2.6)	
Number of children in the home, N (%)			
0	22 (46.8)	69 (45.7)	0.89 ^c^
1+	25 (53.2)	82 (54.3)	
GDM risk factors, N (%)			
Age ≥ 40 years	-	3 (2.0)	
Pre-pregnancy BMI ≥25 kg/m²	-	95 (62.5)	
Newborn’s macrosomia ^e^	-	4 (2.6)	
GDM or signs of glucose intolerance	-	22 (14.5)	
Family history of diabetes	-	88 (57.9)	
Number of GDM risk factors, N (%)			
1	-	97 (63.8)	
2	-	50 (32.9)	
3+	-	5 (3.3)	
Self-reported PA (min/week)			
LPA	197.1 (216)	182.8 (235)	0.56 ^d^
MVPA	179.2 (133)	195.2 (201)	0.88 ^d^
Total PA	376.3 (288)	378.0 (330)	0.96 ^d^
**Characteristics at the seven-year follow-up**			
Age (years)	37.0 (5.5)	37.8 (4.6)	0.34 ^b^
Weight (kg)	63.6 (7.2)	77.7 (15.8)	<0.001 ^b^
Body mass index, BMI (kg/m^2^)	22.9 (2.6)	28.1 (5.7)	<0.001 ^b^
Working status, N (%)			
Fulltime	21 (44.7)	101 (66.4)	0.007 ^c^
Part-time, unemployed, student, maternity leave	26 (55.3)	51 (33.6)	
Marital status, N (%)			
Married	38 (80.9)	120 (78.9)	0.78 ^c^
Not married	9 (19.1)	32 (21.1)	
Number of children in the home, N (%)			
1	5 (10.6)	15 (10.0)	0.90 ^c^
2+	42 (89.4)	135 (90.0)	
Self-reported PA (min/week)			
LPA	295.5 (207)	212.1 (193)	0.005 ^d^
MVPA	206.3 (179)	161.9 (120)	0.33 ^d^
Total PA	501.7 (319)	373.9 (234)	0.012 ^d^
Accelerometer-based PA (% of awake wear time/day)			
SB	71.2 (7.9)	73.9 (6.1)	0.042 ^d^
LPA	18.6 (5.0)	16.8 (4.1)	0.029 ^d^
MVPA	10.1 (3.9)	9.2 (2.9)	0.25 ^d^
**Children’s characteristics, N (boys %)**	47 (55.3)	152 (53.3)	
Age (years)	6.6 (0.5)	6.5 (0.6)	0.33 ^b^
Height (cm)	122.7 (5.5)	123.0 (6.0)	0.82 ^b^
Height for age z-score ^f^	0.11 (0.9)	0.37 (1.1)	0.18 ^b^
Weight (kg)	24.2 (3.6)	24.7 (4.8)	0.82 ^d^
Weight for age z-score ^f^	0.05 (0.9)	0.23 (1.0)	0.31 ^b^
BMI (kg/m^2^)	16.0 (0.2)	16.2 (0.2)	0.56 ^b^
Overweight or obese ^f^	6 (14.6)	31 (21.8)	0.31 ^c^
Accelerometer-based PA (% of awake wear time/day)			
SB	59.0 (5.4)	58.3 (5.9)	0.50 ^d^
LPA	23.1 (3.5)	22.9 (3.2)	0.61 ^d^
MVPA	17.8 (3.3)	18.8 (4.1)	0.19 ^d^

Means (standard deviation) or frequencies (%). PA, physical activity; LPA, light-intensity PA; MVPA, moderate-to-vigorous-intensity PA, SB, sedentary behavior. ^a^ GDMRno = no risk factors for gestational diabetes; GDMRyes = have at least one gestational diabetes risk factor. ^b^ *T*-test ^c^ Chi-square test ^d^ Mann–Whitney U-test ^e^ ≥ 4500 g in any earlier pregnancy. ^f^ According to Saari et al. [23].

**Table 2:** Linear regression analysis of longitudinal relationships between change in self-reported physical activity (PA) (min/week) from baseline to the seven-year follow-up and demographic factors at baseline among women with and without gestational diabetes risk.

	Light-Intensity PA ^c^	MVPA ^c^	Total PA ^c^
	B (95% CI)	B (95% CI)	B (95% CI)
GDMRno ^a^, unadjusted, N = 199	69.0 (−19.4, 157)	60.5 (−2.15, 123)	130 (18.6, 240) *
GDMRno, adjusted ^b^, N = 195	88.2 (20.0, 156) *	41.0 (−6.46, 88.4)	130 (41.9, 217) **
Age	−8.93 (−14.9, −2.92) **	−3.20 (−7.37, 0.98)	−11.8 (−19.6, −4.08) **
Pre-pregnancy BMI	4.02 (−1.97, 10.0)	−1.81 (−6.02, 2.39)	2.48 (−5.28, 10.2)
Education (Reference basic or secondary school)			
Polytechnic	25.3 (−40.0, 90.7)	39.9 (−6.58, 84.6)	60.9 (−23.7, 146)
Academic	13.2 (−59.0, 85.4)	28.8 (−21.3, 78.8)	41.9 (−51.0, 135)
Working status (Reference full day)			
Part-time, student, unemployed, maternity leave	12.4 (−47.8, 72.5)	6.22 (−34.7, 47.1)	13.3 (−63.6, 90.3)
Children in the home (Reference at least one)			
Not at all	−75.1 (−135, −14.9) *	16.8 (−23.6, 57.3)	−63.9 (−140, 12.4)

^a^ GDMRno, mothers with no risk factors for gestational diabetes. ^b^ Adjusted for age (continuous), BMI (continuous), education (Academic/polytechnic/basic or secondary school), working status (fulltime/part-time, being a student, unemployed, or on maternity leave), number of children under seven years (none/at least one) at baseline, and baseline value of the outcome (continuous). ^c^ PA, physical activity; MVPA, moderate-to-vigorous physical activity; CI, confidence interval. * *p*-value < 0.05; ** *p*-value < 0.01; *** *p*-value < 0.001.

**Table 3:** Linear regression analysis of cross-sectional relationships of sedentary behavior and PA (% of awake wearing time) with demographic factors at seven years after the delivery among women with and without gestational diabetes risk.

	Sedentary Behavior	Light-Intensity PA ^c^	MVPA ^c^
	B (95% CI)	B (95% CI)	B (95% CI)
GDMRno ^a^, unadjusted, N = 199	−2.68 (−4.84, −0.53) *	1.78 (0.36, 3.21) *	0.90 (−0.14, 1.94)
GDMRno, adjusted ^b^, N = 195	−0.57 (−2.90, 1.76)	0.40 (−1.12, 1.91)	0.17 (−1.02, 1.36)
Age	0.03 (−0.17, 0.23)	−0.03 (−0.15, 0.10)	−0.001 (−0.10, 0.10)
Current BMI	0.20 (0.03, 0.37) *	−0.11 (−0.22, 0.00)	−0.09 (−0.18, −0.01) *
Education (Reference basic or secondary school)			
Polytechnic	1.62 (−0.55, 3.80)	−0.90 (−2.31, 0.51)	−0.72 (−1.83, 0.38)
Academic	4.25 (1.83, 6.66) ***	−2.99 (−4.56, −1.42) ***	−1.26 (−2.48, −0.30) *
Working status (Reference full day)			
Part-time, student, unemployed, maternity leave	−2.03 (−3.97, −0.10) *	1.74 (0.49, 3.00) **	0.29 (−0.69, 1.27)
Children in the home (Reference at least two)			
One	−1.24 (−4.18, 1.71)	0.77 (−1.15, 2.69)	0.47 (−1.04, 1.96)

^a^ GDMRno, mothers with no risk factors for gestational diabetes. ^b^ Adjusted for baseline education (Academic/polytechnic/basic or secondary school) as well as for age, BMI, working status (fulltime/part-time, being a student, unemployed or on maternity leave), and number of children under seven years (one/at least two) at seven years after delivery, and additionally, for awake wearing time of the accelerometer. ^c^ PA, physical activity; MVPA, moderate-to-vigorous physical activity; CI, confidence interval. * *p*-value < 0.05; ** *p*-value < 0.01; *** *p*-value < 0.001.
